# Patient experience of spontaneous intracranial hypotension (SIH): qualitative interviews for concept elicitation

**DOI:** 10.1186/s41687-023-00625-4

**Published:** 2023-08-15

**Authors:** Timothy J. Amrhein, Molly McFatrich, Kate Ehle, Michael D. Malinzak, Linda Gray, Peter G. Kranz, E. Hope Weant, Christina K. Zigler

**Affiliations:** 1 Department of Radiology, Duke University Medical Center, Box 3808, Durham, NC 27710 USA; 2 Department of Population Health Sciences, Duke University School of Medicine, Durham, NC USA

**Keywords:** Spontaneous intracranial hypotension, CSF leak, Headache, Concept elicitation, Qualitative interview

## Abstract

**Background & objectives:**

Spontaneous intracranial hypotension (SIH) is an underdiagnosed and debilitating condition caused by a spinal cerebrospinal fluid (CSF) leak. Although SIH can lead to substantial morbidity and disability, little data exists about patients’ perspectives. Without hearing directly from patients, our understanding of the full experience of having SIH is limited, as is our ability to identify and use appropriate patient-reported outcome measures (PROMs) within clinical care and research. The purpose of this study was to conduct qualitative interviews with confirmed SIH patients to fully describe their experiences and identify relevant concepts to measure.

**Methods:**

Patients were recruited from an SIH specialty clinic at a large, U.S.-based healthcare center. Patients undergoing an initial consultation who were ≥ 18 years old, English-speaking, met the International Classification of Headache Disorders-3 criteria for SIH, and had a brain MRI with contrast that was positive for SIH were eligible to participate. During semi-structured qualitative interviews with a trained facilitator, participants were asked to describe their current SIH symptoms, how their experiences with SIH had changed over time, and the aspects of SIH that they found most bothersome. Analysts reviewed the data, created text summaries, and wrote analytic reports.

**Results:**

Fifteen participants completed interviews. Common symptoms reported by patients included headache, tinnitus, ear fullness/pressure/pain, and neck or interscapular pain. Patients reported that their symptoms worsened over the course of their day and with activity. The most bothersome aspect of SIH was disruption to daily activities and limits to physical activities/exercise, which were severe. With regard to symptoms, the most bothersome and impactful included physical pain and discomfort (including headache), as well as fatigue.

**Conclusions:**

Patients reported a diverse set of symptoms that were attributed to SIH, with devastating impacts on functioning and high levels of disability. Researchers considering use of PROMs for SIH should consider inclusion of both symptom scales and aspects of functioning, and future work should focus on evaluating the validity of existing measures for this patient population using rigorous qualitative and quantitative methods in diverse samples. Additionally, these data can be used to assist clinicians in understanding the impacts of SIH on patients.

**Supplementary Information:**

The online version contains supplementary material available at 10.1186/s41687-023-00625-4.

## Background

Spontaneous intracranial hypotension (SIH) is an underdiagnosed condition caused by a spinal cerebrospinal fluid (CSF) leak that leads to substantial morbidity and disability [1–3]. Patients with SIH classically suffer from orthostatic headaches (i.e. headaches that are worse when upright and relieved with recumbency) as well as various cranial nerve related symptoms including diplopia, tinnitus, disequilibrium, and cognitive dysfunction [4]. There has been marked increase in the number of recognized cases of SIH in the past decade and it is now considered an important treatable cause of secondary headaches [5]. The efficacy of various treatments for SIH (i.e. epidural blood patching, surgery, venous embolization) are poorly established due to a paucity of prospective studies and the absence of any randomized controlled clinical trials [6]. The field is now pivoting toward these critical studies in order to establish consensus recommendations and to define a clear treatment algorithm. In order for these studies to be meaningful, patient reported outcome measures (PROMs) that adequately capture the spectrum of SIH patient symptoms and disability must be identified or developed.

To be appropriate for use in clinical care and research contexts, PROMs for SIH should be anchored concretely in the patients’ experiences, capture the concepts that are most salient to patients, and supported by rigorous validity evidence [7–9] The first step in this process is to elicit these concepts from patients using rigorous qualitative methods [7]. To our knowledge, there are no PROMs with validity support for use in SIH, and to date, no PROMs have been developed specifically for SIH. A literature review on the efficacy of treatments for SIH reveals that the vast majority of studies report patient reported outcomes (PROs) subjectively without using formal PROMs, report symptoms based on the clinician’s impression, or do not describe outcome assessment methods at all [10]. The few studies that do include established PROMs use a visual analogue scale (VAS), which assesses only pain and is therefore woefully inadequate for capturing the range of symptoms and the spectrum of disability in SIH. Other currently available headache PROs may not capture the spectrum of what SIH patients experience given the considerable variability in presentation ranging from headache, visual disturbance, auditory changes, memory loss, etc [1]. To our knowledge, there are no prior studies that attempt to understand SIH by directly asking patients about their experience. This is important because a physician derived and reported understanding of SIH may differ from the lived experience of patients, and we are not able to measure what matters to patients without this understanding.

The purpose of this study was to conduct concept elicitation interviews with SIH patients to understand the key concepts that are relevant for understanding the symptoms and experiences of patients with SIH [7]. We plan to use the information from these qualitative interviews to develop or identify appropriate measures for adults with SIH that are grounded in the patient experience.

## Methods

This is a prospective, qualitative concept elicitation study, using a narrative research approach [7]. The aims of concept elicitation are to understand the experience of the patient, identify common and uncommon symptoms that patients associate with SIH, and to document the terminologies used by patients when describing their experiences.

### Participants & setting

Patients who were 18 years of age or older, English-speaking, met the International Classification of Headache Disorders-3 (ICHD-3) criteria for SIH, had a brain magnetic resonance imaging (MRI) with contrast that was positive for SIH (e.g. pachymeningeal enhancement, brain sagging, venous distension sign), and had not been previously treated at our institution were eligible to participate [11]. As part of standard clinical care, patients were contacted over the phone by the Neuroradiology Nurse Practitioner (NP) in order to gather clinical history, review imaging, and to determine suitability for a visit to Duke. A board certified Neuroradiologist with at least 6 years of experience specializing in the diagnosis and treatment of SIH reviewed all patient data and imaging to confirm the diagnosis. Once eligibility was assessed, the patient was contacted by a member of the qualitative study team to consent and schedule the interview. The Duke Institutional Review Board deemed this study exempt from human subjects research.

### Data collection

Three qualitative interviewers, with an average of 5 years of qualitative interview experience, conducted one-on-one interviews with participants via a semi-structured interview guide (Supplementary Material 2), with interviewers given freedom to ask follow up probes of their own design, and at their own discretion. Participants were asked to describe their current SIH symptoms, how their experiences with SIH have changed (e.g. worse vs. better), and the aspects of SIH that they found most bothersome. Based on clinical knowledge of SIH, a list of potential symptoms was also included in the interview guide as possible probes [4]. If participants did not spontaneously discuss a symptom on the list, the interviewers would probe on the specific symptom. Finally, participants were asked about what first prompted them to seek treatment, what they did to manage their SIH symptoms, and their goals for upcoming treatment. All interviews were conducted over Zoom and audio recorded with participant permission. Interview recordings were transcribed via a third-party transcription service, and then quality checked by one of the analysts upon return. Participants were given a $25 gift card as remuneration.

### Data Analysis

In addition to the audio files and interview transcripts, interviewers took detailed notes during the interview. In their notes, they reported on any challenges the participant had with speech (e.g. difficulty forming words, slurring of speech, stuttering), difficulty finding words, perceived attitudes about the questions asked, and interview duration. The interviewer conducting the interview then used all of these data (i.e. audio files, transcripts, interview notes) to complete individual interview text summaries shortly following completion of the interview using a standardized debriefing form [12, 13]. Text summaries were used to evaluate the data at the individual participant level, and included descriptions of the symptoms mentioned, dominant themes and other findings. Interviewers met bi-weekly during data collection to discuss the findings from the interviews, including symptom experiences of the participants and emerging ideas.

Once data collection was finished and all the individual interview text summaries were complete, two analysts, who were also interviewers, worked together to review the data across all participants. Analysts worked together to organize data by subtopic, based on the interview guide. From there, analysts divided sections of data, read through the data, and generated notes on these data as themes emerged. For the symptom list, analysts reviewed participant descriptions of their symptoms throughout the course of the interview and then grouped those experiences in a symptom list. Then the two analysts worked together to write analytic reports summarizing the emerging themes. Throughout the analysis process, the two analysts met frequently to discuss findings and provide feedback on each other’s writing. Final analytic reports were reviewed and approved by the study team. Based on these qualitative interviews of the SIH patient experience as well as the symptom list, target concepts were identified and recommendations for potential useful PROMs were generated in tabular format.

## Results

Interviews occurred between August 2021 and January 2022. Of the 17 patients contacted, 15 agreed to participate and two patients did not return our attempts to contact them.

*Demographics*. The 15 individuals included 6 men and 9 women with diagnosed SIH who were on average 51 years old (*std* = 10 years). Most participants were white and all had at least a high school degree or equivalent (Table 1). Two reported that they were on disability leave from work, with 4 reporting full time employment, 4 reporting part-time employment, and 4 being retired. Five participants had no previous treatment for their SIH, with 9 participants reporting prior epidural blood patches, with the average number of patches being 2 (range: 1–5). About half of the participants (7/15) had a previous history of headaches prior to developing SIH.

**Table 1:** Demographic information for the 15 adult participants with confirmed SIH.

Variable	*n* (%)
Female	9 (60.0)
Race/Ethnicity	
White	14 (93.3)
African American or Black	1 (6.7)
Highest grade in school	
High school degree or equivalent	2 (13.3)
Some college university	3 (20.0)
College/university degree	7 (46.7)
Post-graduate degree	3 (20.0)
Annual Household Income	
I prefer not to answer/I do not know	5 (33.3)
Less than $20,000	1 (6.7)
Between $20,001 and $40,000	1 (6.7)
Between $60,001 and $80,000	1 (6.7)
More than $80,001	7 (46.7)

*Symptoms.* Headache pain was reported by all participants when asked to describe their experience with SIH (Table 2). The majority of patients described their headache as including pain and pressure at the back of their head. Words like “pulling”, “pushing”, and “squeezing” were used to describe the pain and pressure, and “sharp” or “shooting” to indicate severe pain. One participant (ID: sc10) compared it directly to their experiences with typical headaches, *“I would say it’s more a pressure. And I experience it – initially it was kind of all over and more like a typical headache. But after time and I started to curb my activity, it just becomes more of a pressure thing.”*

**Table 2:** Frequency^^^ of symptoms reported by 15 adult participants with SIH.

Symptom as described by the patient (medical term)	Total (n)	Unprompted^1^ (n)
Head pain/headache	15	15
Ringing in ears / muffled hearing (tinnitus)	13	5
Ear fullness / pressure, and/or pain	9	6
Neck pain	9	5
Interscapular pain	9	3
Nausea	9	2
Fatigue	8	2
Cognitive/memory issues	8	1
Dizziness / light headedness / sense of imbalance (disequilibrium)	7	5
Difficulty finding words (anomic aphasia)	6	0
Head pressure	6	6
Sensitivity to sound (hyperacusis)	6	0
Sensitivity to light (photophobia)	6	1
Blurry vision / double vision (diplopia)	3	0
Hearing loss	3	1
Impaired speech (e.g., difficulty forming words, stutter)	2	2
Runny nose or liquid drainage (rhinorrhea)	2	1
Vomiting	2	0
Loss of mobility	1	1
Low back pain	1	1
Difficulty swallowing (dysphagia)	1	1
Eyeball pain (ophthalmalgia)	1	1
Sensitivity to smell (hyperosmia)	1	0

^1^Indicates the subset of total patients who offered this symptom themselves after the first open-ended question. The total *n* includes all patients who endorsed the symptom before/after specific prompting from the interviewer. ^^^Frequency counts only include participants who were “sure” about experiencing each symptom (some participants reported uncertainty about experiencing certain symptoms or the source of the symptom)

Unprompted by the interviewer, the next most frequently listed symptoms were head pressure (which was discussed separately from head pain by patients) and ear pressure. Blurry vision, difficulty remembering things, word finding difficulty, sensitivity to light and sound were all symptoms that multiple participants endorsed, but almost no participants endorsed those symptoms until the interviewer listed them as an option (Table 2). Other less common symptoms included impaired speech (e.g., difficulty forming words, stutter), runny nose or liquid drainage (rhinorea), vomiting, loss of mobility, low back pain, difficulty swallowing (dysphagia), eyeball pain (ophthalmalgia), and sensitivity to smell (hyperosmia).

*Factors affecting symptom rating and attributes.* Several participants stated that their symptoms got worse as the day went on. To try and manage the pain and other symptoms, most participants would lie down or be still as often as they could. Participants used phrases like, if they “catch it early” it is easier to manage, but if they “push it” (e.g., don’t lay down, sit down, etc.) the symptoms (especially head pain and pressure) become unmanageable.

Another factor that affected perceived severity was the presence of an accompanying symptom. For example, dizziness with headache, or nausea with headache led the participant to classify the headache symptom as severe or intense, but days with only headache pain were classified as moderate. For example, one participant (ID: sc06) said, *“If I had the spinning, then I would say it was severe but I haven’t had the spinning actually in a couple weeks; and that’s when it’s bad. But I’m also at a point where there’s things I don’t do and especially won’t do in the afternoon because of the possibility of the dizziness.”*

*Most Bothersome Aspects of SIH.* When discussing the most bothersome aspect of living with SIH, participants talked about the disruption to daily activities caused by the painful and uncomfortable symptoms they experienced, including headache, fatigue, and nausea (Table 3). For example, people mentioned being frustrated that they could not run to the store on a whim or physically pick up their children. Similarly, other participants identified the most bothersome aspect of SIH as limits on their ability to be physically active and to exercise. Participants described having to “dial things back” or limit their activities in order to prevent pain episodes. Most participants described needing to lie down or sit down several times a day in order to manage the head pain and alleviate the head pressure. Moreover, two participants mentioned the social impact of not being able to do certain activities because those activities could cause them pain. Three participants said that their SIH limited their ability to interact with their children, restricting their physical movement so they could not hold their children or impacting their ability to participate in school activities (e.g. see quote from participant #sc04 in Table 3).

**Table 3:** Descriptive quotes from 15 confirmed SIH patients describing the most bothersome aspects of living with SIH.

ID	Quotes
Sc01	*“I mean, yeah. The biggest problem is the pain. It might be just everything else is from the pain. When you’re in a lot of pain, you don’t wanna be in a bright light situation. You wanna be in the dark where it’s like you can just kinda focus inward and let it pass.”*
Sc02	*“Used to go to church a lot, but of course, COVID stopped that, but then again, if it did start up, I have a hard time with that – being able to go somewhere and sit for a while. Every now and then, it flares up, and it’s like, “Oh, I can’t do this.” So, I guess that comes with the good days and the bad days.”*
Sc03	*“Well, it’s more of an aggravation to me, I guess, because I want to do certain things, and I know I can’t do it without getting a painful headache.”*
Sc04	*“Yes, it’s just hard to not –to feel like I can’t do those things because I don’t want to have to deal with that pounding, intense headache”* *“I mean, I would say I have that sensation at least at some point every day. I’ve got three kids, so I’ve got a lot of bending and picking up and cleaning, and I have an infant, so physically exerting myself is like I just don’t have a choice. So, I would say every day there is something that I do within the day that tells me that’s too much, you know, because I get that sensation. I wouldn’t say it is as often or lasts as long as it did previously. Like when I was pregnant, it would put me down for a couple of hours, and I could only get up in like 20, 30-minute increments and have to go lay back down. So, it’s not as intense or frequent as it was, but I also know how to mitigate it better now, I guess.”*
Sc05	*“Particularly through the peak of it, I just couldn’t work a full work day, and would need to come home and take a nap in the middle of the day. There’s the asterisk that I was having stomach problems, too, as to exactly what was the origin of the fatigue, or was it both, I don’t know. It’s hard to say. But having to literally change how I lived my life, cut my work hours back, was a pretty major impact on my life. And so kind of a tie between that and the headaches.”*
Sc06	*“The dizziness. Bar none”*
Sc07	*“Not being me. Just not being able to pick up and run out to the store, not being able to go on a field trip with my son because I’m afraid I’m gonna have the pain, just not able to do things for others.”*
Sc08	*“Not being able to do things because of the pain. It’s pretty annoying.”*
Sc09	*“The intensity. For the most part, today I have a 1 out of 10 headache. Not bad at all. And a 1 out of 10 dizziness, vertigo kind of thing. But not bad at all. And I just go through the day and it’s perfectly fine and I don’t have any problem. The only problem is when it gets worse…But those are the most bothersome times, when it really interferes with my life.”*
Sc10	*“For me having the curbed activity. Not being able to play tennis. […] Being concerned that if I play tennis that I’m gonna get a headache or make things worse or maybe my ringing will come back. So, just not being able to be fully active. And concerns that if I don’t get this treated that something else might happen. You know. That I might have a stroke or something like that.”*
Sc11	*“It’s really just, I think, the impact of the quality of life and just feeling kinda crummy. The symptoms of profound fatigue and the head pressure just make you not want to do anything. And it also makes me grouchy, so I feel like I’m kinda grumpy. And I don’t complain to my family, so if I don’t feel well I don’t tell them I don’t feel well. I just am probably not the nicest person because I don’t feel well. I don’t like to complain about it because there’s nothing I can do…about it. I need to do something because it does impact my quality of life.”*
Sc12	*“The headache…It just doesn’t stop. It just ruins my day, and night.”* *“Well, I just can’t get out of my own way. Like, we were trying to take a walk up our road, and I just –My husband had to go back home and get the car. I just wanted to lay down in the road and go to sleep. I have no energy. I have no strength at all.“*
Sc13	*“Assuming all my eye problems are because of this, and I am for the purpose of this conversation, I would say all the eye issues. The other things I can work around, but if I’m potentially losing vision, color vision, I can’t see. I have to close my eyes by 6:00 every night. I mean, that’s completely life-limiting. So, the pain, pressure, the eye situation, it’s a handicap. I mean, I am handicapped by it.”*
Sc14	*“Being limited on what I can do because I’ve always been a go getter. I like getting stuff done. I like that feeling of accomplishment at the end of the day, but this has just stopped me in my tracks. I just can’t do what I used to could do, so that to me has been the most frustrating.”*
Sc15	*“The headaches, the pressure, and then the whole cognitive. I know I keep saying all of those, and I don’t know if you want me to choose one, but if I had to choose one to say, “Okay, you can only fix one, you can only solve one,“ oh my god, I think I would be like – I don’t know, ‘cause they’re all pretty bad. The cognitive is more all the time kind of, so to speak. The focus or even, again, I wake up and I can’t pronounce California. I can kinda see it, but I can’t pronounce it. That right there is very frustrating.* *The headaches, the pressure – the headaches, again, are not all day, for the most part, unless I have a bad day and then I have a headache all day. But it’s not that headache that is just like something punches you in the head, type of thing. But those right there are where I ask my partner, “Can you check on me in the middle of the night, please?“ So, those are very painful, and I feel like something’s gotta be happening that is not good. This is not good. I don’t know–”*

*Expectations for treatment.* Most patients stated they hoped treatment could improve the headache severity and pain intensity. Many have the expectation that the pain and accompanying symptoms will completely go away with treatment. A few participants stated they were unsure if all symptoms would go away, but they were hopeful that treatment could at least help with the headache severity, and they stated they could live with mild headaches if there was no choice.

*“I get migraines, and people ask me, “Well, try your migraine medication.” It doesn’t work because during this time, I have had migraines, and I’ve taken my medication, and that alleviates my migraine, but it does not alleviate the pain and the pressure in the back of my head.”* - sc02

*“Yeah. Well, I hope I can help someone else. And yeah I’m just excited because I was thinking probably even 50 years ago, I bet there wasn’t even treatment for something like this. So, I feel really fortunate that they have a way to hopefully treat this and control it.“* – sc10

*Experience with diagnosis.* Nine participants reported that they received treatment for another condition before they were diagnosed with SIH. These included: sinus infections, migraines, allergies, sarcoidosis, TMJ, stress, and psychological issues, as well as side effects of pre-existing conditions such as back problems or pregnancy. Participants described waiting for months or even years between the initial onset of their symptoms and their diagnosis or treatment. Of the 13 participants who reported a timeframe, 4 reported that they were treated within about 3 months of initial symptoms, 5 reported they were treated within 6 months to a year of the initial onset of symptoms, and 4 reported it took over a year from the onset of symptoms to their SIH diagnosis, with one participant reporting that they were still waiting (over a year) for treatment as of their interview date. The longest time to diagnosis of those we spoke to was over 2 years. Nearly all participants (11/15) reported that their initial symptom was a headache or migraine. Two participants were diagnosed with SIH as a result of a scan for another condition. One participant was trained as a medical professional and was therefore able to advocate for a diagnosis after recognizing the symptoms, despite their doctor’s opinion that the symptoms were stress-related. Another reported that their headaches started after a car accident, and they were able to easily access care and receive a diagnosis quickly because they were friends with a neurologist. One participant described having to beg to be seen by their spouse’s neurologist out of desperation for answers. Once participants were diagnosed, they described the comfort that their SIH diagnoses brought; with one (ID: sc07) saying: *“It was a relief to know it was not all literally in my head, that they had findings, that there is something going on, and they can see it, that it’s not just stress.”* While another reported being “thrilled” to finally have a diagnosis.

### Potentially useful PROMs

A number of measurable concepts were identified as meaningful to patients diagnosed with SIH. For each concept, existing measures with published validity evidence were identified as potentially useful for patients with SIH (Table 4).

**Table 4:** Measuring what matters to patients with spontaneous intracranial hypotension (SIH): Recommended concepts to target with Patient-Reported Outcome Measures (PROMs) based on qualitative interviews with patients

Meaningful patient experience identified via qualitative interviews	Outcome of Interest	Existing patient-reported outcome measures (PROMs) that may be useful in measuring the outcome of interest^1^
Primary Symptom - pain	Headache pain* (intensity/frequency)	Headache Impact Test (HIT-6); PROMIS Pain Intensity; Numeric Rating Scale; Pain, Enjoyment, General Activity (PEG) Scale Assessing Pain Intensity and Interference
Other symptoms - varied	List of common and uncommon symptoms associated with SIH (presence/absence)	Due to unique nature of SIH, the development of an SIH-specific symptom battery could be considered
Other symptom – fatigue	Fatigue (intensity)	PROMIS Fatigue
Other symptoms – cognitive impairment	Cognitive impairment	PROMIS Cognitive Functioning
Functioning	Pain interference*	Pain, Enjoyment, General Activity (PEG) Scale Assessing Pain Intensity and Interference
Functioning	Physical Functioning*: Impact to daily activities and limits to physical activities/exercise	PROMIS Physical Functioning
Functioning	Social Role Functioning/Participation	PROMIS Ability to Participate in Social Roles and Activities
Headache characteristics	Headache pain characteristics (frequency)^2^: Improve when lay down, worsen when bent over, worsen when lift heavy objects, worsen as day progresses	The development of SIH-specific items could be considered.

(1) Please note, there are currently no PROMs with validity support for use with patients with SIH. The listed measures are provided as a potential guide for clinicians and researchers, but their appropriateness is tied to any intended context of use and setting. It is generally advised that a minimal amount of cognitive testing and psychometric evaluation be conducted before measures are used with any new patient population. (2) Items could be used to differentiate between SIH headache and headache due to rebound intracranial hypertension. (3) Outcomes that are included in the National Institutes of Health HEAL Common Data Elements for studies evaluating treatment for acute and chronic pain are marked with a *

## Discussion

This article represents, to our knowledge, the first description of patient experiences using qualitative methods to elicit meaningful concepts directly from patients with ICHD-3 confirmed SIH. As expected, headache was the primary symptom in SIH with all patients reporting the presence of a headache without prompting. SIH patients also described multiple additional concerning symptoms. Tinnitus and ear fullness were the next most common after headache, followed by neck and interscapular pain, nausea, fatigue, cognitive dysfunction, disequilibrium, hyperacusis, photophobia and diplopia, as well as many others. It is important to note that eliciting these symptoms required prompting in many cases, and that some patients conceptualized ‘worse’ days as those when they had additional symptoms alongside their headache (e.g. dizziness, nausea).

The list of symptoms associated with SIH that patients provided in the qualitative interviews align with and expand on those previously reported in the literature. In a systematic review and meta-analysis, D’Antona et al. found that headache was the most common symptom in SIH patients (present in 97% of patients) [4]. The reported non-headache SIH symptoms also align with this work, although our study was more exhaustive and included additional symptoms not previously captured such as interscapular pain, word finding difficulty, photophobia, hearing loss, impaired speech, rhinorrhea, ophthalmalgia, and hyperosmia. Movement disorders (e.g. ataxia, bradykinesia) were not reported by our patient cohort, but are rarely reported in the SIH literature, and are typically represented in the form of single case reports [14–16]. It also is important to note that patients utilized lay terms when describing their symptoms (see examples in Table 2); any symptom PROM that is developed should utilize terminology that patients can understand.

Universally, SIH patients reported considerable disability and a substantial impact on their daily function. In a cross-sectional online survey of SIH patients, Cheema et al. [17] also found substantial reductions in quality of life in SIH patients using the EQ-5D-5 L measure [18] and that the headaches due to SIH resulted in a very severe impact on functioning based on the Headache Impact Test (HIT-6) [19]. The qualitative results in our study are consistent with these findings. This prior report also found that greater than 25% of SIH patients had lost their job secondary to the resultant disability, which is greater than the rate found in our cohort (n = 2) [17]. This discrepancy may be due to the smaller sample size in the present study and the fact that 26.7% (n = 4) were already retired. In addition to functioning, patients in our cohort described impacts on social/role functioning as well as impacts on daily activities that were often considerable.

This study formally and qualitatively documents the patient experience of SIH, but also generates two important conclusions that can to help guide future measurement strategies. First, patients should be prompted to capture the extent and breadth of variably present symptoms in SIH. In the absence of prompting, it is likely that some symptoms that are concerning to patients and that may be helpful in differentiating SIH from other causes of headache will remain uncaptured. Second, patient disability was considerable and impacts on function were high. As a result, any patient-centered measurement strategy for SIH should capture not only symptom severity but also the downstream effects on quality of life such as disruptions in social, emotional, and physical functioning.

Based on the results from our qualitative interviews, we have identified a number of concepts that are measurable and meaningful to patients (Table 4). While identifying what is important to patients is vital for any measurement strategy, decisions about which specific PROMs to utilize for SIH will ultimately be dependent on the proposed context of use [20]. Currently, the field would benefit from measures that track patient status over time within a clinical setting, as well as highly accurate and sensitive measures to evaluate treatment benefit. An additional use of measures would be to differentiate between SIH and other diagnoses. Thus, our recommendations (Table 4) include existing measures that may be useful for these purposes, with a focus on measures following a rigorous process for development (e.g. PROMIS®) and/or those listed within common data elements (e.g. National Institutes of Health Heal Initiative) [21]. In addition to the list provided, we suggest that clinicians and researchers also consider screening for depression and anxiety in these patients [21]. Please note, further cognitive debriefing and psychometric evaluation are needed to support the choice of specific measures; this work is currently underway.

Establishing a diagnosis of SIH can be challenging given the protean symptom profile which can overlap with that expected for other diagnoses. For example, the classic presenting symptom in SIH, an orthostatic headache, is not always present in SIH patients and can be seen in the setting of other diagnoses such as cervicogenic headache and postural orthostatic tachycardia syndrome [22, 23]. As a result, greater than 90% of SIH patients are initially misdiagnosed, delaying curative intervention and compounding disability through prolonged disease courses and unnecessary treatments [24]. Qualitatively documenting the spectrum of symptom presentation in SIH will allow for future research focused on specific symptoms that may allow for the differentiation between these often confused clinical diagnoses.

Although the demographics of the included population in this study generally align with those reported in the prior SIH literature, [4] our sample was relatively affluent. Given that the patient cohort was derived from those presenting to a major SIH treatment center, this likely reflects the requirement for considerable travel for treatment in most cases, including across state lines. Ideally, future studies would further expand upon our work to include a broader and more diverse subset of the SIH population. Another potential limitation of this study is the stringent inclusion criteria that required not only a confirmed ICHD-3 SIH diagnosis but also a brain MRI that was positive for SIH findings. It is well known that some patients with ICHD-3 confirmed SIH have a normal brain MRI [22]. There may be yet unknown differences in the SIH patient experience and symptom profile between those with a positive brain MRI and those where it is negative. Finally, given the prevalence of memory dysfunction in the SIH population, the inability of patients to recall some of their experiences could introduce bias when trying to capture the full spectrum of disease, a potentially pervasive problem that is not limited solely to this study.

## Conclusion

SIH is a debilitating condition that is increasingly recognized as a treatable cause of secondary headaches. However, the conduct of important studies and the development of treatment guidelines have been hampered by the lack of validated PROMs for SIH, without which it is difficult to determine the comparative efficacy of different treatments. This study represents the first critical step in establishing such PROMs by reporting the SIH patient experience using qualitative concept elicitation interviews. Future work should build upon this effort to develop new measures or to determine the appropriateness of existing measures which should capture both the severity of patient symptoms as well as the impact of SIH on patient function and the extent of their resultant disability.

### Electronic supplementary material

Below is the link to the electronic supplementary material.

Supplementary Material 1: Standards for Reporting Qualitative Research (SRQR)

Supplementary Material 2: Interview Guide

## Data Availability

Not applicable.

## Declarations

### Disclaimer

The views expressed in this submitted article are those of the authors and do not represent an official position by the funder or institution.

## Abbreviations

SIH: Spontaneous intracranial hypotension
CSF: Cerebrospinal fluid
PROM: Patient-reported outcome measure
VAS: Visual analogue scale
MRI: Magnetic resonance imaging
NP: Nurse practitioner
HIT-6: Headache impact test
ICHD-3: International classification of headache disorders-3 criteria
