# Supplementary material for: Patient experience of spontaneous intracranial hypotension (SIH): qualitative interviews for concept elicitation
Source: J Patient Rep Outcomes. 2023 Aug 15;7:82. doi: 10.1186/s41687-023-00625-4 (PMC10427581; doi:10.1186/s41687-023-00625-4)
Supplement: Supplementary file 2 — Supplementary Material 2: Interview Guide [file 41687_2023_625_MOESM2_ESM.docx]

**Interview Guide**

*Date of interview: ___________________*

*Participant ID number: __________________*

*Interviewer’s name: __________________*

*Interview Start Time (24-hour clock): ______:______*

**SECTION: Read Introduction Page to Participant [5 minutes]**

As a reminder, the goal of this study is to learn about your experience with Spontaneous Intracranial Hypotension (SIH) and how it impacts your daily life. During the interview, we’ll start by discussing your symptom experiences and how they impact different activities and your quality of life.

The interview will take about an hour. Your participation is completely voluntary. You can choose to take a break at any time or stop the interview entirely.

Please know that there are no right or wrong answers. I am interested in your thoughts and experiences.

I would like to audio record the interview because I want to make sure I don’t miss any of your comments. If you don’t want the interview audio recorded, I will take detailed notes during the interview instead. As a reminder, the recording will be stored securely and eventually destroyed after we publish the study’s findings.

**Do I have your permission to record this interview?**

**□Yes** **□No**

Do you have any questions for me so far about the interview?

**START AUDIO RECORDER (if participant gave permission to do so)**

**This is [INTERVIEWER NAME] and I’m with participant [ID number] on [Date].**

**SECTION: Concept Elicitation [25 minutes]**

1. **How would you describe your SIH in your own words?**

*[Note to interviewer: Symptoms and physical effects may include headache, neck pain, interscapular pain, nausea, vomiting, sensitivity to light and/or sound, changes in vision, sense of imbalance, ringing in the ears, changes in hearing, fatigue, memory issues, word-finding difficulty]*

1. **Tell me about your current SIH symptoms.**

**Probes:**

- [Note to interviewer: probe on all symptoms mentioned]
- Tell me about what [symptom] feels like?
- Would you say [symptom] was mild, moderate, or severe? Why?
- What time frame are you thinking of? How would your answer change if I asked you to think about the past week? 30 days? Longer?
- How did you incorporate…
  - How often do you have <symptom>?
  - How severe or intense is the symptom?
  - Tell me about the timing of symptom (day or night) in your answer, if at all? Is there a pattern to your [symptom] (e.g. does it happen mostly at night, in the morning?)
- [For **headache** specifically] Where do you feel the pain during your headache? *[Note to interviewer, may include: Left side, right side, forehead, etc.]*
- *What does the* ***headache*** *pain feel like?*
- [For patients with a separate history of headaches] How is your headache pain different from the pain related to SIH/CSF leaks?

1. **Please tell me about how your experience with SIH fluctuates or changes, if at all.**

**Probes:**

- - What triggers your worst symptoms, if anything?
  - What makes your symptoms better?
- How do you describe your good days with SIH? Your bad days?
- How does your body position affect your symptom?

1. **How does SIH affect your life?**

**Probes:**

- - What about your daily activities?
  - What activities do you avoid?

1. **What aspects of SIH do you find most bothersome?**

**Probes:**

- What do you find second most bothersome? Why?
- What things do you worry about related to your SIH?

**Section: Treatments [10 minutes]**

Thank you for answering those questions for me. Now, during this part of the interview, I’d like to talk about your experience with different treatments for SIH. I do not need to know about medicines you have taken, but I’d like to learn more about your experiences with medicine or treatment.

1. **What first prompted you to seek treatment?**
   - How did you know it was different than just a migraine/headache?
2. **What do you do to manage your symptoms currently?**
3. **What would successful treatment of SIH look like to you?**

**Probes:**

- - What aspects of your SIH would you want the medicine/treatment to address? (frequency, intensity, or something else)
  - If there was a treatment available for SIH, what is the one most important thing you’d hope it could improve about your condition?

**Those are all of the questions that I have for you. Is there anything else that you’d like to share with me that you think is important for us to know?**

**Thank you very much for your help today.**

**Do you have any other questions? Thank you again. I will be mailing you your gift card/ClinCard as thanks for participating in this study.**
